# Development of a cutaneous fistula following hepatic cystic echinococcosis

**DOI:** 10.1186/s40064-015-1327-6

**Published:** 2015-09-22

**Authors:** Serhat Akay, Nazif Erkan, Mehmet Yildirim, Huriye Akay

**Affiliations:** Emergency Medicine Clinic, Izmir Research and Training Hospital, Izmir, Turkey; General Surgery Clinic, Izmir Research and Training Hospital, Izmir, Turkey

## Abstract

Hydatid cyst is an infectious disease characterized by cysts formed primarly within the gastrointestinal tract by echinococci. Hepatic hydatid disease, which is the most common form, remains asymptomatic until complications occur. In this report, we present an 80 years old patient who presented with a hepatic hydatid cyst which fistulized to the abdominal skin into the Emergency Department. Computed tomography of the abdomen showed inactive grade 5 cyst. Drainage without removal of the cyst failed to reveal active disease but the microbiological examination showed Klebsiella pneumonia that was sensitive to ampicillin–sulbactam as the causative agent. The treatment of the cyst with a combination of surgical and medical treatment was the successful treatment of Hepatic Hydatid Disease presenting with a cutaneous fistula.

## Background

Cystic echinococcosis is a parasitic disease caused by the genus *Echinococcus,* affecting primarily the hepatobiliary, respiratory, and less frequently, the central nervous system. The cysts resulting from the parasitic infection remain asymptomatic for many years, and the initial presentation is usually due to the complications arising from fistula formation. The hepatobiliary system is the most common site of the fistulae, while cutaneous fistulae are rare (Sayek et al. 2004). Here, we report a rare clinical case of cystic echinococcosis presenting as a cutaneous fistula with a secondary bacterial infection.

## Case

An 80-year-old man was admitted to the Emergency Department (ED) with right upper quadrant abdominal pain and a cutaneous lesion with white, purulent discharge that appeared a week ago in the same region. The patient’s medical history was notable for an operation for the removal of a liver abscess of unknown etiology fifteen years ago; otherwise, no other chronic illnesses were present.

**Fig. 1:**
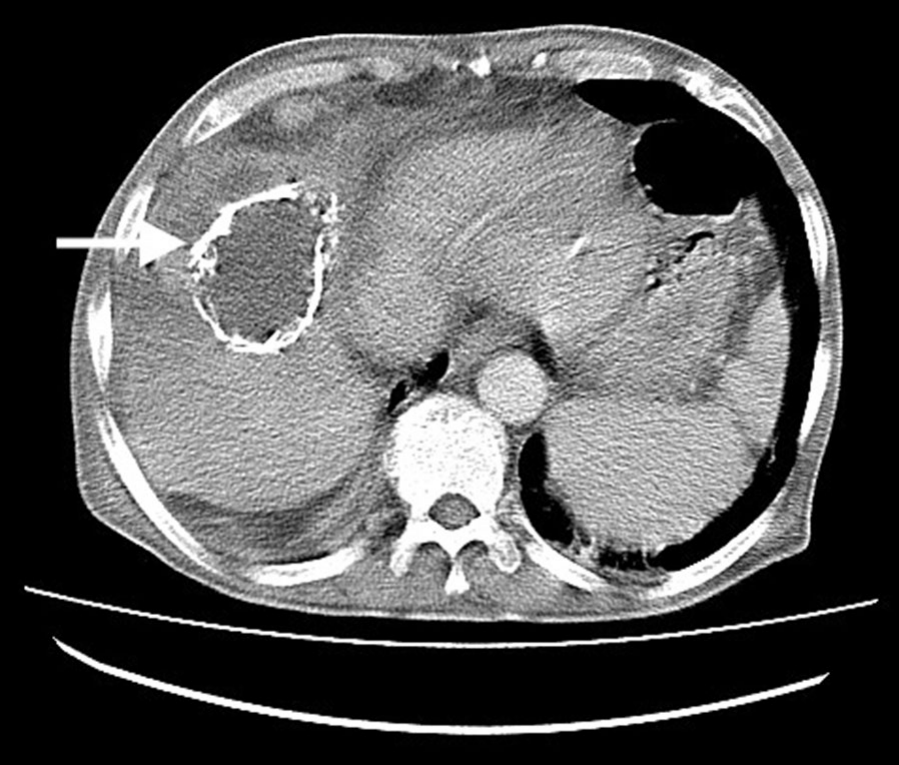
Calcified cystic lesion in the right lobe of the liver (*arrow* indicating the cyst)

During physical examination, the abdominal wall was tender on palpation, and the examination of the lesion in the upper right quadrant revealed a fistula opening with white fluid discharge. The physical examination of other systems was normal. The abdominal ultrasonography performed in the ED showed a cystic mass with a diameter of 6 cm in the seventh and eighth segment: the cyst had a calcified membrane and a comet-tail artifact that indicated the presence of air. The cystic lesion appeared to protrude to the subcapsular area and subcutaneous tissue: these findings were highly suggestive of a fistula. The biochemical values and complete blood counts were within normal limits. An abdominal computed tomography with intravenous contrast revealed a single, calcified, membranous lesion in segments 8 and 4A with a fistula to the abdominal anterior wall (Fig. 1). In addition, the increased subcutaneous density within the cyst was noted. Surgical drainage by subcostal incision under local anesthesia resulted in approximately 100 mL of purulent material. The cyst was not excised due to the advanced age of the patient. Nonetheless, bradycardia and hypoglycemia that developed during the postoperative recovery necessitated the patient to be admitted to the intensive care unit for further treatment. Importantly, the analysis of the purulent material from the cyst failed to confirm an *Echinococcus* infection; however, *Klebsiella pneumonia* that was susceptible to most commonly used antibiotics was cultured from the purulent material, upon which a 14-day course of ampicillin-sulbactam was initiated. The patient underwent repeated abdominal ultrasonography, which failed to show a recurrence of similar cystic lesions in the abdomen. The patient was discharged 7 days after hospitalization and was followed at the outpatient clinic. The evaluation of the patient at an outpatient setting by ultrasound imaging 6 months after the procedure did not show a recurrence of the cyst.

## Discussion

Human echinococcosis is a zoonotic infection caused by one or more of the four known strains of *Echinococcus* parasites. The parasitic life cycle includes both herbivores and carnivores, including dogs and sheep; however, humans may become infected as intermediate through the ingestion of infected organs. Cystic echinococcosis presents as multiple cysts throughout the body, and is epidemic in the Mediterranean and certain parts of Africa and Central Asia. However, increased transcontinental travel has led to an increase in cystic echinococcosis in nonepidemic regions as well.

In humans, the asymptomatic initial infection is followed by the symptomatic phase, the type and severity of which depends on the location, size, number and mass effect of the cyst/s. For example, a noncomplicated cyst can cause symptoms secondary to its local affects such as the enlargement of the affected organ and impingement on surrounding tissue. When liver is infected, increased pressure exerted by the cyst can produce symptoms of jaundice or abdominal pain, whereas lung involvement can present as hemoptysis, dyspnea or chronic cough.

A common complication of the cystic echinococcosis is the cystic rupture and subsequent allergic reaction to the parasitic material, which ranges from urticaria to anaphylaxis depending on the volume of the leaked material. Other commonly observed complications include the compression of, or communication with, the biliary tree and secondary echinococcosis (Sayek et al. 2004).

According to the Ghabli classification established by The World Health Organization-Informal Working Group in Echinococcus (WHO-IWGE), a type 5 cyst is defined as calcification of the cystic membranes, as determined by imaging characteristics, similar to our observations in our patient. Typically, a type 5 cyst is presumed inactive; however, in rare cases, an abscess with cutaneous fistula/e can occur in the calcified cavity, and typically presents with symptoms that were also observed in our patient. Importantly, previously reported cases of type 5 cysts include cutaneous fistulae with cystic material drainage in the absence of concomitant bacterial infection. In contrast, the microbiological evaluation of aspirated material in our patient did not reveal any active parasites, but was positive for *Klebsiella pneumonia* (Salerno and Cracolici 2006; Yakan et al. 2009; Korwar et al. 2011). To date, there is no consensus on the best treatment modalities for cystic echinococcosis is still lacking, partially due to the lack of prospective studies and the rarity of the disease. A systematic review of treatment approaches for both complicated hepatic and disseminated cystic echinococcus suggest pericystectomy with drainage of the calcified hepatic cyst; however, this advice is based only on one retrospective analysis, i.e., a level IV evidence, and warrants further research (Dziri et al. 1999).

Lewall and McCorkell have classified the rapture of hepatic echinococcal cysts into three (Lewall and McCorkell 1986). The contained rupture is defined by the containment of endocyst by the intact pericyst. Communicating rupture involves the drainage of cyst material into the bronchioles and biliary tract that are incorporated into the pericyst. Direct rupture, as in our case, occurs when the endocyst and pericsyt rupture with the subsequent leakage of cyst components, leading to complications such as anaphylactic shock and secondary bacterial infections (Kjossev and Teodosiev 2013).

*Klebsiella pneumonia* infections are typically localized to the lower respiratory, urinary, and biliary tracts; surgical wound sites are also commonly infected. Predisposing factors include alcohol abuse, diabetes, malignancy, liver parenchymal disease and chronic obstructive pulmonary disease. The rate of reported pyogenic *Klebsiella pneumonia* liver abscesses are increasing (Lederman and Crum 2005). In our case, the patient’s history of a liver abscess and advanced age might have contributed to the development of a liver abscess in a grade 5 hepatic echinococcosis cyst that was superinfected with *Klebsiella pneumonia*.

Based on our experience, we recommend that the drainage of the infected material with antibiotic treatment for the secondary bacterial infection may be an alternative approach in cases when surgical removal of the cyst may not be achievable due to confounding factors.

## Conclusion

In summary, cutaneous fistulae originating from hepatic echinococcal cysts is a rare complication of active cysts. Grade 5 cysts are considered inactive; however, the formation of fistulae to abdominal wall and superinfection by a second microorganism, as that occurred in our patient, can be successfully treated with drainage. This approach appears to be feasible with no risk of recurrence in cases where the cyst cannot be removed. Finally, *Klebsiella pneumonia* may be the cause of abscess formation in a subset of patients.
